# Supplementary figures and images for: Factors influencing TB treatment interruption and treatment outcomes among patients in Kiambu County, 2016-2019
Source: PLoS One. 2021 Apr 6;16(4):e0248820. doi: 10.1371/journal.pone.0248820 (PMC8023511; doi:10.1371/journal.pone.0248820)

TREATMENT INTERRUPTION TRACING FORM


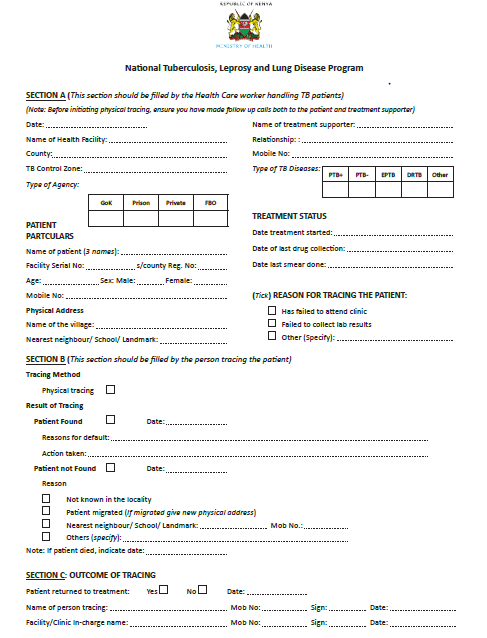

Supplement: S1 File — (DOCX) [file pone.0248820.s001.docx]
