# Supplementary material for: Factors influencing TB treatment interruption and treatment outcomes among patients in Kiambu County, 2016-2019
Source: PLoS One. 2021 Apr 6;16(4):e0248820. doi: 10.1371/journal.pone.0248820 (PMC8023511; doi:10.1371/journal.pone.0248820)
Supplement: Attachment — Submitted filename: Letter to the editor.docx [file pone.0248820.s003.docx]

Dear Editor,

Thank you for your review. We have addressed the comments as highlighted in blue. Thank you.

1.) Please ensure that your manuscript meets PLOS ONE's style requirements, including those for file naming. The PLOS ONE style templates can be found at

<https://journals.plos.org/plosone/s/file?id=wjVg/PLOSOne_formatting_sample_main_body.pdf> and

<https://journals.plos.org/plosone/s/file?id=ba62/PLOSOne_formatting_sample_title_authors_affiliations.pdf>

 Thank you. Yes, this was checked; the manuscript conforms to the PLOS One authors submission guidelines

2.) You indicated that a waiver of consent was granted for your study,; however, you have not indicated whether the institutional review board waived the need for ethical approval. We understand that the framework for ethical oversight requirements for studies of this type may differ depending on the setting and we would appreciate some further clarification regarding your research. Could you please provide further details confirming from your institutional review board or research ethics committee (e.g., in the form of a letter or email correspondence) that ethics review was not necessary for this study? Please include a copy of the correspondence as an "Other" file."

 Yes there was a waiver; we have included a section on ethical consideration on page 7 and attached the ethical approval letter as an additional file (S2).

3.) We suggest you thoroughly copyedit your manuscript for language usage, spelling, and grammar. If you do not know anyone who can help you do this, you may wish to consider employing a professional scientific editing service.  

Whilst you may use any professional scientific editing service of your choice, PLOS has partnered with both American Journal Experts (AJE) and Editage to provide discounted services to PLOS authors. Both organizations have experience helping authors meet PLOS guidelines and can provide language editing, translation, manuscript formatting, and figure formatting to ensure your manuscript meets our submission guidelines. To take advantage of our partnership with AJE, visit the AJE website (http://learn.aje.com/plos/) for a 15% discount off AJE services. To take advantage of our partnership with Editage, visit the Editage website ([www.editage.com](http://www.editage.com/)) and enter referral code PLOSEDIT for a 15% discount off Editage services.  If the PLOS editorial team finds any language issues in text that either AJE or Editage has edited, the service provider will re-edit the text for free.

Upon resubmission, please provide the following:

- The name of the colleague or the details of the professional service that edited your manuscript
- A copy of your manuscript showing your changes by either highlighting them or using track changes (uploaded as a *supporting information* file)
- A clean copy of the edited manuscript (uploaded as the new *manuscript* file)

 Thank you this has been checked, the grammar and the spelling has been checked.

4.) Please provide further details on sample size and power calculations.

 There was no formal sample size calculation as this was a retrospective study, however the final sample included in the study was assumed to have enough power to detect a difference between the outcome and explanatory variables. This is explained in page 6.

5.) In the statistical analysis section, please clarify which confounders or variables were accounted for in your statistical models.

There is a section added on study variables on page 7. All variables were considered important in explaining the outcome of interest and were therefore adjusted for in the cox proportion model.

6.) Please include additional information regarding the data extraction tool used in the study and ensure that you have provided sufficient details that others could replicate the analyses. For instance, if you developed the data extraction tool as part of this study and it is not under a copyright more restrictive than CC-BY, please include a copy, in both the original language and English, as Supporting Information, or include a citation if it has been published previously.

We used a treatment interrupters tracer form which is a Ministry of Health approved tool to capture the participant’s details and reasons for treatment interruption; this has been indicated in page 5. This tool has been attached as an additional file (S1)

7.) We note that you have indicated that data from this study are available upon request. PLOS only allows data to be available upon request if there are legal or ethical restrictions on sharing data publicly. For information on unacceptable data access restrictions, please see <http://journals.plos.org/plosone/s/data-availability#loc-unacceptable-data-access-restrictions>.

In your revised cover letter, please address the following prompts:

a) If there are ethical or legal restrictions on sharing a de-identified data set, please explain them in detail (e.g., data contain potentially identifying or sensitive patient information) and who has imposed them (e.g., an ethics committee). Please also provide contact information for a data access committee, ethics committee, or other institutional body to which data requests may be sent.

b) If there are no restrictions, please upload the minimal anonymized data set necessary to replicate your study findings as either Supporting Information files or to a stable, public repository and provide us with the relevant URLs, DOIs, or accession numbers. Please see <http://www.bmj.com/content/340/bmj.c181.long> for guidelines on how to de-identify and prepare clinical data for publication. For a list of acceptable repositories, please see <http://journals.plos.org/plosone/s/data-availability#loc-recommended-repositories>.

We will update your Data Availability statement on your behalf to reflect the information you provide.

 The data set has been uploaded as an additional file, S3.

8.) Your ethics statement should only appear in the Methods section of your manuscript. If your ethics statement is written in any section besides the Methods, please move it to the Methods section and delete it from any other section. Please ensure that your ethics statement is included in your manuscript, as the ethics statement entered into the online submission form will not be published alongside your manuscript.

Thank you. The Ethics section has been added to the methods section page 7.

9.) Please include your tables as part of your main manuscript and remove the individual files. Please note that supplementary tables (should remain/ be uploaded) as separate "supporting information" files

 The tables have now been included as part of the main manuscript.

 The STROBE guidelines for cohort studies informed the design and reporting for this study. This has been added as additional file, S3.

The manuscript strongly concludes that men are prone to treatment interruption compared to women yet the sample population included 68% men. Wouldn't including more men bias your study findings? We would appreciate this discussion in the limitation section of your manuscript.

Majority of treatment interrupters in our study were men, this might have introduced a selection bias in the population included in our study. This statement has been added in page 14.

In addition the peer reviewer has highlighted some changes that warrant your attention. These include a mismatch between statistical figures in the abstract and manuscript, encouragement to reference the latest WHO TB reports and Kenyan National Leprosy and TB program reports, misreporting of acronyms, consistency in the use of HR or aHR among other minor comments.

Thank you, this has been corrected. We used the current reports as at the time of developing this study.

Yours sincerely,

Evelyn Kimani
